# *“Quality teaches you how to use water. It doesn’t provide a water pump”*: a qualitative study of context and mechanisms of action in an Ethiopian quality improvement program

**DOI:** 10.1186/s12913-023-09341-7

**Published:** 2023-04-19

**Authors:** F Procureur, AS Estifanos, DW Keraga, AK Kiflie Alemayehu, NW Hailemariam, J Schellenberg, H Magge, Z Hill

**Affiliations:** 1 Institute for Global Health, University College London, Guilford St, London, WC1N 1EH UK; 2 Department of Reproductive, Family and Population Health, School of Public Health, Addis Ababa University, Addis Ababa, Ethiopia; 3 Institute for Healthcare Improvement, Addis Ababa, Ethiopia; 4 Department of Disease Control, London School of Hygiene and Tropical Medicine, Keppel Street, London, WC1E 7HT UK; 5 Brigham and Women’s Hospital, Division of Global Health Equity, 75 Francis Street, Boston, MA 02115 USA

**Keywords:** Quality improvement, Quality improvement collaboratives, Context, Mechanisms, Implementation, Low-and middle-income countries, Ethiopia

## Abstract

**Background:**

Quality improvement collaboratives are a common approach to bridging the quality-of-care gap, but little is known about implementation in low-income settings. Implementers rarely consider mechanisms of change or the role of context, which may explain collaboratives’ varied impacts.

**Methods:**

To understand mechanisms and contextual influences we conducted 55 in-depth interviews with staff from four health centres and two hospitals involved in quality improvement collaboratives in Ethiopia. We also generated control charts for selected indicators to explore any impacts of the collaboratives.

**Results:**

The cross facility learning sessions increased the prominence and focus on quality, allowed learning from experts and peers and were motivational through public recognition of success or a desire to emulate peers. Within facilities, new structures and processes were created. These were fragile and sometimes alienating to those outside the improvement team. The trusted and respected mentors were important for support, motivation and accountability. Where mentor visits were infrequent or mentors less skilled, team function was impacted. These mechanisms were more prominent, and quality improvement more functional, in facilities with strong leadership and pre-existing good teamwork; as staff had shared goals, an active approach to problems and were more willing and able to be flexible to implement change ideas. Quality improvement structures and processes were more likely to be internally driven and knowledge transferred to other staff in these facilities, which reduced the impact of staff turnover and increased buy-in. In facilities which lacked essential inputs, staff struggled to see how the collaborative could meaningfully improve quality and were less likely to have functioning quality improvement. The unexpected civil unrest in one region strongly disrupted the health system and the collaborative. These contextual issues were fluid, with multiple interactions and linkages.

**Conclusions:**

The study confirms the need to carefully consider context in the implementation of quality improvement collaboratives. Facilities that implement quality improvement successfully may be those that already have characteristics that foster quality. Quality improvement may be alienating to those outside of the improvement team and implementers should not assume the organic spread or transfer of quality improvement knowledge.

**Supplementary Information:**

The online version contains supplementary material available at 10.1186/s12913-023-09341-7.

## Introduction

Efforts to improve quality of care in low and middle-income settings include interventions such as Quality Improvement Collaboratives (QICs) [1–3]. This approach is based on narrowing the gap between what we know and do, by providing health facilities with a structure to learn from experts, from each other and to act on areas suitable for change. Facilities, or departments within facilities, form an improvement team who, with the support of a mentor, implement small scale rapid tests of changes to address a specific topic through ‘Plan-Do-Study-Act’ cycles. Several teams form a collaborative which meets at ‘learning sessions’ to learn improvement methods, facilitate peer learning, and accelerate improvement towards a common aim [4].

While the implementation of QICs has been growing in low and middle-income settings, there have been criticisms that impacts have been limited and unpredictable, in part because implementers overlook the mechanisms of change and the influence of the implementation context [5–7]. A better understanding of what works and why is key if implementers are to improve the design, implementation, evaluation and transferability of the QIC approach [5, 8]. For example, an understanding of the role of context could allow QICs to be contextually adapted or implementers could make efforts to improve the context, or readiness, prior to implementation [5].

Knowledge of the role of mechanisms and context in QICs comes mainly from high-income settings and from studies of hospital care [5, 7]. These studies have proposed that QICs increase capacity, generate a learning and collaborative culture, increase data quality and data use, and create healthy competition and peer pressure. Contextual factors that may be important include leadership characteristics; organizational and individual capabilities/characteristics; data and technical infrastructure; relationships; readiness for change; core values and ideologies, and how well the quality improvement (QI) topic aligns with existing priorities, strategies, targets and incentive systems [5, 7]. This paper aims to add to the sparse knowledge base on the mechanisms through which QICs work and the role of context in resource limited settings through a qualitative study of large-scale implementation in Ethiopia.

## Methods

### Quality improvement collaboratives in Ethiopia

The Ethiopian Ministry of Health has quality and equity as a core pillar of its Health Sector Transformation Plan [9], with high level engagement and investment in QI [10]. In 2017, the Ministry of Health partnered with the Institute for Healthcare Improvement (IHI) to develop a National Health Care Quality Strategy to build QI capacity at all health system levels and to test and scale a QIC approach [4, 10]. The aim was to accelerate change in maternal and neonatal health by improving the coverage and quality of antenatal (ANC), delivery and postnatal care (PNC) in five regions through 26 *woreda* (district) level collaboratives. Hospitals formed maternal and neonatal QI teams and each health centre formed a QI team with its health posts. Representatives of the QI teams across all facilities in each *woreda* met at four quarterly collaborative learning sessions. In between learning sessions facilities tested one or more change ideas with the support of regular visits from QI mentors who provided clinical and QI support [10]. Learning session facilitation and mentorship was done jointly by IHI project officers and *woreda* health staff, with a gradual transition to *woreda* staff during the intervention [11]. The QIC also utilized a childbirth checklist to measure the quality of delivery management for three ‘clinical bundles’ (Supplementary file 1) and as an entry point for clinical mentorship. Prior to the formation of the QIC, IHI worked with each facility to improve the quality of their routine data by engaging leadership and by validating reported data against registers, tally sheets and medical records. A focus on data quality was continued during the QIC.

### Study setting

We collected data in Amhara and Oromia regions – the most populous in the country. In each region we selected one *woreda*, which had no unusual characteristics compared to others in the regions in terms of economy, ethnicity and health system support, and had completed their final learning session within the last two months. To ensure the safety of the data collectors we excluded areas that had active localized conflicts at the time of data collection. Within each *woreda* we selected the *primary* hospital, one health centre that the IHI project officer considered had better-functioning QI and one considered to be functioning less well. Functioning was defined as QI teams being more active and QI activities being more integrated into the facility workflow. We refer to these as higher and lower performing facilities; higher functioning may not equate to high functioning as it was defined in relative not absolute terms. The study was conducted at a time of political instability and civil unrest linked to political reform, youth protests and ethnic tension, which resulted in violent clashes and mob attacks, which were sometimes aimed at researchers and health workers [12, 13].

### Qualitative data collection

Qualitative data were collected between June and August 2019 by five experienced interviewers who received a 5-day training which included piloting the instruments. In-depth interviews were conducted in the local language and included free flowing questions to allow for unanticipated themes and theoretically driven questions informed by mechanisms identified in the literature (See Table 1) [5, 7].

**Table 1:** Topics covered in-depth interviews with facility staff

Interview topic	Example of content
The QI team and the learning sessions	- Structure and function of the QI team - Who is selected to attend learning sessions and how - Perceptions and experiences of the learning sessions
The mentors	- Role of mentors - Perceptions of and experiences with the mentor - Level of support received and its importance
The change ideas	- Selection of change ideas - Implementation successes and challenges - Any reluctance or resistance to implementation
Data and monitoring	- Monitoring of change ideas - Challenges and successes in collecting and using data
The facility	- Communication, openness and leadership - Team work - Infrastructure and equipment - Accountability, recognition and reward - Any changes in the above related to QI

In each facility we interviewed between 5 and 10 staff. We first interviewed the facility manager, maternal and the child health (MCH) focal and/or neonatal intensive care unit (NICU) focal and/or antenatal care (ANC) focal person and the health information technician (HIT) – all of whom usually attended learning sessions. The IHI project officer and the initial participants then identified other staff involved in QI, including Health Extension Workers (HEWs) from the health posts. Potential participants were then approached by the study team. Participants were purposively selected to include both those who attended and did not attend QI learning session. We also interviewed all the IHI and *woreda* mentors that supported the two collaboratives on their role and experiences with the QIC.

Respondents gave written informed consent and all agreed to be interviewed. Interviews were audio recorded and transcribed into English by the interviewers as soon after the interview as possible. Data analysis began during data collection through regular reflexive meetings where we discussed preliminary themes, gave interviewers feedback on their transcripts and interview technique, and modified questions and probes as needed. Interviews were then coded inductively in NVIVO for each facility in turn by the lead and last author. Finding were compared and contrasted across facilities. A theme was classed as a mechanism if it was directly related to the intervention (e.g. increased knowledge of the importance of quality), and as a contextual factor if it was a pre-existing characteristic of the facility (e.g. its infrastructure) that influenced QI implementation. To align with the existing literature, we separated our mechanisms into between and within-facility mechanisms [5, 7].

### Quantitative data collection

Intervention facilities collated monthly data on seven core coverage (percentage of mothers who attended the facility for a particular service) and quality (percentage of mother attending the facility who received recommended care) indicators. We extracted data for three coverage and one quality indicator (See Table 2) that used Health Information System data collected on all women who attended the facility; other indicators came from the childbirth checklists which were used inconsistently across facilities. We constructed p and u control charts with the centre line calculated across the data set unless there was evidence of special cause variation, that is atypical variation in the charts, or when the chart remained unstable for 20 or more data points. In these cases, the centre line was phased. The baseline was defined from the completion of initial data validation and ended between the first and second learning sessions when we would expect change ideas to start impacting on the indicators. Control chart rules were then applied [14, 15].

**Table 2:** Indicators of progress

Indicator	Numerator	Denominator*
***ANC coverage*** Percentage of women who attended at least 4 ANC visits (ANC4)	Number of women who attended at least four ANC visits (range 1-115 women)	Number of expected pregnancies (range 43–226 pregnancies)
***ANC quality*** Percentage of pregnant women attending ANC tested for syphilis	Number of pregnant women tested for syphilis during their first ANC visit (range 0-395 women)	Number of pregnant women who attended first ANC visit (range 11–395 women)
***Delivery management coverage*** Percentage of births attended by skilled health personnel (SBA)	Number of births attended by skilled health personnel (range 3-143 deliveries)	Number of expected deliveries (range 43–226 deliveries)
**PNC coverage** Percentage of women who attended postnatal care 48 h after delivery (PNC-48)	Number of postnatal visits within 48 h of delivery (range 0-103 visits)	Number of expected deliveries (range 43–226 deliveries)

*Calculated using a nationally standardized system guided by the Central Statistics Agency (CSA) census data

Special cause variations were identified with the implementation team to assess if they were likely to be intended or unintended.

## Results

In this section, we first present information on the respondent characteristics (Table 3), and the study setting and study facilities, including a summary of QI indicators for each facility. We then present the QI mechanisms that were identified from the data and finally contextual factors that influenced whether the mechanisms were triggered or not.

**Table 3:** Respondents and sample size (2 hospitals and 4 health facilities)

	Attended QI learning sessions	
Respondents	Yes	No
Facility manager	6	0
MCH/ANC/NICU focal person (midwives/nurses)	8	0
Health Information Technician (HIT)	3	2
Midwife	3	4
Nurse	0	5
Health officer (HO)/doctor	2	0
Laboratory focal person	0	3
Pharmacy focal person	0	1
Health Extension Workers	7	4
Mentors	2 IHI 5 *woreda* health staff	0
**Total**	**36**	**19**

### Study setting

Table 4 shows the study facility characteristics which were either observed, extracted from the interviews or provided by IHI. Each facility was labelled with a code to maintain anonymity corresponding to region (A or B) and level of performance (1 = high and 2 = low). The two high performing facilities had reasonable access to water, electricity, drugs and equipment and had strong leaders and good teamwork. The poor performing facilities had poor access to water, inconsistent electricity and issues with drugs and equipment, they had weaker leaders and a culture of working as individuals and staff conflicts. As each sampled woreda only had one hospital these were not selected based on their level of performance. Both hospitals had access to water and electricity but respondents reported issues with buildings and equipment and with leadership and teamwork. Respondents from the three facilities in region B reported that political instability and civil unrest in their catchment area had affected both service delivery and uptake.

**Table 4:** Summary of study facilities

	Health centres				Hospitals	
	A1: Higher performing	A2: Lower performing	B1: Higher performing	B2: Lower performing	A3	B3
**Description of area**						
*Location*	Village near main *woreda* town, easy access by road	Remote, highland village, difficult access by rough road	Remote village with sparse population, access by flat road	Remote, highland village with sparse population, difficult access by rough road	*woreda* main town easy access by a flat road	*woreda* main town easy access by a flat road
**Infrastructure**						
*Water*	Mains	Purchased by the bucket	Water tank	Purchased by the bucket	Mains	Mains
*Electricity*	Mains	Solar panel: not always functioning	Generator: usually has fuel	Generator: often without fuel	Mains	Mains
*Infra-structure and supplies*	Necessary infrastructure, supplies and equipment	Insufficient buildings and a lack of equipment (e.g. beds) and drugs	Insufficient buildings	Insufficient buildings and irregular supplies of drugs and material	Insufficient buildings and some lack of equipment	Insufficient buildings and a lack of equipment
**Staff and HR information**						
*Leadership*	Facility manager strong and active	Facility manager often absent but broadly supportive of QI	Facility manager strong and active	Facility manager not fully supporting QI	Facility manager not fully supporting QI	Facility manger not supporting QI and inexperienced
*Teamwork*	Good cooperation, team-work and motivation	Staff work as individuals with staff conflicts	Good cooperation, team-work and motivation	Staff work as individuals with staff conflicts, but some degree of openness reported	Staff work as individuals with staff conflicts	Staff work as individuals with staff conflicts
**Political instability and civil unrest**						
	None	None	A key challenge	A key challenge	None	A key challenge
**Signals of improvement in coverage**						
	Improvement in ANC4, SBA and PNC-48 Processes stable	Improvement in ANC4 Degradation followed by a rebound to initial levels for SBA and PNC-48 Processes unstable	Degradation in all indicators Processes unstable	Degradation in all indicators Processes unstable	Small improvement in ANC4 No improvement in other indicators Processes unstable	Small improvement in PNC-48 No improvement in other indicators Processes unstable

### Quantitative data on changes in the selected core indicators

Health centres in region A showed signals of improved coverage, especially the higher performing facility (see Table 4). Health centres in region B showed degradation, and both hospitals showed signals of a small improvements for one coverage indicator. Coverage of syphilis testing was high at baseline, except in facility A2, and high testing coverage was generally maintained through the intervention period, but there were some months with coverage outside of the control limits in the poorer performing health centres and hospital A3. See Supplementary files 2 and 3 for the control charts for each facility and a summary of the signals of improvement.

### Qualitative data on QI mechanisms and contextual influencers

Described below are the seven mechanisms we identified through which QI worked and the five contextual factors that influenced whether mechanisms were triggered.

### Between-facility mechanisms

#### Modified perceptions of and focus on quality

Learning session attendees reported an increased awareness of the importance of quality, the impact of poor quality on families and had reflected on quality gaps in their facilities. Some respondents reported the sessions changed their perspective on their efficacy for problem solving: *‘Quality is about mind-set, previously we took shortage of water as a problem and refused to work… after quality* [QI] *our attitude changed and we contribute money and buy water’* [B2 HO]. This focus on quality was maintained through the mentors’ visits.

#### Learning from experts and each other

Learning sessions were described as opportunities for learning QI principles, for starting QI processes and for sharing knowledge and experiences: ‘E*veryone shares and takes knowledge and experiences… we have shared our projects and we have also adopted from others’* [A3 MCH focal]. Generally, the sessions were viewed very positively, but some HEWs felt alienated by the language used: *‘The training did not encompass the HEWs…. It was not inviting for us….*’ [B1 HEW4] and other participants struggled with the content as they were invited to only one session: *‘We didn’t get enough understanding…. it’s new for us … they give only highlight’* [B1 midwife2].

#### Competition between facilities

During the learning sessions facility teams presented data on their change ideas, which was described as a *‘competition’*. Having improvement acknowledged by others elicited feelings of pride and ownership and was motivational: *‘When achievements of our plans are presented and we show our success… .I felt like I am the owner of the success. So, I felt proud…. That feedback is the thing that builds us’* [A1 MCH focal]. Presenting no or little impacts was viewed as presenting *‘failure*’, *‘having low performance’* or *‘scoring less’* which was *‘unpleasant’, ’upsetting’*, and *‘shameful’*. For some this served as motivation to improve: ‘*He* [another facility manager] *presented his amazing work and I was upset at myself because I was working in the old way, I haven’t improved anything. After that presentation onwards, I just started to work hard to improve my facility’* [A2 Facility manager]. But, for others the shame and embarrassment inhibited openness: *‘When we are not doing well we feel ashamed to talk in front of people…. I feel bad and I’m not comfortable to talk* [A1 HEW1]. Despite the shame that came from poor performance, respondents described learning sessions as different from their usual *woreda* meetings which were characterised by explicit shaming and blaming.

### Within-facility mechanisms

Within facility mechanisms were all related to mentors whose presence enhanced sustainability, skills, motivation and procurement.

#### New structures and processes

QI resulted in structural changes through the development of a QI team with set process to follow. QI team members who had attended learning sessions described the cycle of selecting problems, understanding the root-cause, designing change ideas, setting targets and monitoring performance. Teams also reported a new appreciation for data through new processes of auditing, analysing and using data. Maintenance of these structures and processes was difficult in facilities where structures were stimulated by the QI mentor, where they were not reinforced by seeing improvement and because of issues such as staff turnover and competing priorities: *‘When we try to arrange the meeting we find only one or two* [QI] *members… some maybe busy… some have left the facility’* [B2 MCH focal]. The level to which QI was shared outside of those who attended learning sessions varied, and where sharing did not occur some staff found the structures and processes alienating: *‘I don’t know how it* [QI] *came or how it is done…. The responsibility of these activities are held by a few individuals… I personally feel distanced’* [A2 pharmacist].

#### Learning and support

Mentors reinforced learning session content and supported QI and clinical skills and problem solving: *‘He* [IHI mentor] *was always close and assisted us in every step…. He is watching us grow… showing us the way’* [A3 MCH focal]. IHI mentors were perceived as more knowledgeable and consistent than *woreda* mentors who themselves reported challenges with their skill level, workload and transport: *‘Honestly speaking, there will be gap* [in mentorship] *as there is gap regarding transportation…. Supportive supervision . will not be adequate*’[A Woreda Mentor]. IHI mentors were praised for their expertise, skills, ability to inspire and motivate and their characters: *‘In one hour he* [IHI mentor] *gives you the information that may take more than five days of training* [A2 QI focal]. The mentoring focused on the MCH department, and those from other departments (e.g. laboratory or pharmacy) sometimes felt unsupported: *‘I don’t know them* [mentors]… *he goes to the midwives…it is better if they go to all departments… We have trained* [attended learning sessions] *but they didn’t do anything to check whether we are implementing’* [A3 HIT].

#### Increased accountability and motivation

Respondents felt accountable to their IHI mentors and wanted to please them and avoid shame. In most cases this was motivational, but in a few cases it resulted in avoidant behaviours: *‘At the time* [the] *mentors come, if the worker did not fill* [the data forms], *then they run away’* [A2 MCH focal]. Inconsistent visits, for example due to access or security issues, were discouraging and impacted QI activities: *‘The activities get delayed when the mentorship gets loose’* [A1 MCH focal]. Respondents in three facilities (B1, B2, A3) felt QI was not sustainable without IHI mentors: *‘If IHI stops their support, QI will also stop…. You can do more when you think someone will come…. the woreda is not supporting us’* [B1 midwife2]. Respondents from the other three facilities also felt *woreda* mentorship would be inadequate, but described QI as institutionalized, internalized and part of daily activities and, although it may decline as IHI phased out, they felt it would continue in some form.

#### Improved procurement

Mentors helped identify gaps in equipment and sometimes advocated for their purchase: ‘*QI did not supply drugs, but it pushed the region to help’* [A1 Midwife1]. Some facilities were able to negotiate the use of budgets for equipment, lobbied the woreda when there was a shortage of drugs or identified that they already had existing equipment: *‘After the training…. we took money and purchased the needed laboratory kit… even there was equipment in the store… no one had opened the store to see’* [A1 Facility Manager].

### Contexual factors

The lower performing facilities and hospitals were characterised by weak leadership, poor teamwork, and poorer infrastructure and resources. In all facilities staff felt the broader health system was unfair as opportunities were based on politics rather than achievement and hard work. Facilities in region B were impacted by political instability and civil unrest. These contextual factors are discussed below:

#### Leadership

Respondents from the higher performing health centres reported strong, supportive and engaged leadership: *‘I am one of the workers…. I am the head but I can clean toilets, the other staff clean with me’* [A1 Facility manager]. These leaders were instrumental in problem solving (e.g. overcoming financial and administrative hurdles and improving procurement), keeping QI on the agenda (e.g. by ensuring meeting were held) and were hands on with QI processes. In these facilities other supportive structures such as Performance Monitoring Teams were more likely to be functioning and QI was sometimes integrated into these existing systems. In the lower performing health centres and the hospitals leadership was described as weak, unsupportive or disengaged. This was attributed to leaders having other priorities, not receiving financial benefit from QI, being inexperienced and not being committed to the facility: *‘If he was present he would have had the chance to correct many things’* [A2 HEW02]…… *‘I am not supporting the team with full capacity, well I don’t have any special incentives’* [B3 Facility manager]. Where leadership was weak facilities were more reliant on the IHI mentors to ensure QI structures and processes were followed: ‘*When the mentor came we meet together and do something…. other than this we haven’t done’* [B2 Midwife1].

#### Teamwork, dialogue and harmony

Strongly linked to the theme of leadership was teamwork in terms of how staff supported each other, shared tasks, and communicated. In the higher performing health centres and one hospital, there was a strong sense of openness, shared responsibility and goals, and co-operation: *‘We have good communication starting from head to the cleaners and guards, we have good communication throughout. We cannot say this is my duty and this one is not; we all participate in everything and speak to each other’* [A1 HIT]. This enabled a shared culture of improvement to develop among the team members, improved morale, and staff were adaptable in their tasks to facilitate change idea implementation. In addition, QI knowledge was more likely to be passed to others within and outside the QI team: *‘Our staff are like one person, they think as one person, even when there is new comer he will be part of the team immediately, everybody knows about QI’* [A1 Facility manager], which meant that staff alienation and turnover was less problematic. In facilities with poor teamwork staff were more rigid in their roles: *‘We all concentrate on our day to day job…. No one took QI as part of his job…*[or] *personal duty…. We think of QI as additional task’* [B2 HIT] and QI remained the remit and responsibility of the QI team. Other staff lacked knowledge and ownership of QI, so were more resistant to change and QI was more susceptible to staff turn-over. In the poorer performing facilities and hospitals respondents reported staff conflicts which reduced teamwork, increased turnover and became a distraction: *‘I didn’t assist the team or check on their progress while they write QI projects…. Since we* [head and deputy] *were fighting, we focus on how to find faults on each other. We totally forgot about our responsibilities’* [A2 Facility manager].

Where present, teamwork was reported to have existed before QI implementation: *‘It existed before QI training; there is communication, working together and if there is gap in our work we can discuss how to fill that gap’* [A1 Midwife1]; and where absent not to have been influenced by QI or influenced only in the short term: *‘Most of the time they* [staff] *work alone rather than in a team… …. at the beginning of QI we functioned well as a team… … but now everything is forgotten’* [A3 NICU focal].

#### Infrastructure, resources and workload

The higher performing facilities had better infrastructure, equipment and supplies. These, along with workforce, budget and topography influenced teams abilities to implement QI projects and to provide quality services in general: *‘It needs some supply inputs… to bring quality… there are activities that need resources… if they can’t do anything* [about it], *it* [quality] *will only be found on paper’* [B3 CEO]. Respondents felt many of these structural challenges were unsolvable and blamed the government, the poverty of the country and external decision makers: ‘*The MCH has only one bed it is very short and uncomfortable for the mother to sleep on that. To solve this, material support is needed…… The government is the one to be blamed… There is no road, no water supply and inadequate power supply…. Quality works theoretically… quality teaches you how to use water. It doesn’t provide a water pump to bring the water’* [A2 pharmacist]. These issues also affected morale. In the facilities where QI was not taken as a shared responsibility, with little knowledge transfer outside of the QI team, implementation was also more strongly affected workload and by staff turnover.

#### Political instability and civil unrest

Facilities in region B were affected by political instability and civil unrest, which resulted in safety concerns, high stress levels, staff desertion and temporary migration, lack of transport, lack of supplies and temporary closure of facilities: ‘*People were concerned on how to save their own life rather than working… we were thinking of our own safety not the job’* [B1 HEW4]*…. ‘This year there was chaos…. it was big obstacles for all activities* [B1 HEW3]…… *‘Only one midwife was available…. no glove, lidocaine and any other drug because the staff locked their office and go somewhere… the instability was very difficult’* [B2 HEW1]. QI structures and processes were reduced or stopped and mentor visits were missed or done via the phone. The instability also had a strong impact on utilization as families choose to delivery at home or in a safer area, with trust slow to return: *‘We talk to the mothers, and they respond that you can’t save my life when some conflict happened there* [in the facility]*’* [B1 MCH focal]. Support for the facility was also reduced: *‘Kebele leaders were not cooperative… they give priority to issues of political stability, they want to establishing peace first…. mothers’ health was not their issue at the time* [B2 Facility manager]*’* and some community leaders advised non-utilization of facilities. The impact was similar for the higher and lower performing facilities, both of which reported that it was difficult to reinitiate QI once their facilities began to function again.

#### Perception of system justice

In most facilities staff felt that opportunities in the facility, or within the health system more broadly, were unfair in that that hard work was not recognised, and decisions on trainings, transfers and promotions was politically motivated. For staff at the more remote facilities with poor infrastructure a lack of fair opportunities was felt more acutely, leading to low morale and lower motivation to implement QI.

### Inter-connections and feedback loops

**Fig. 1:**
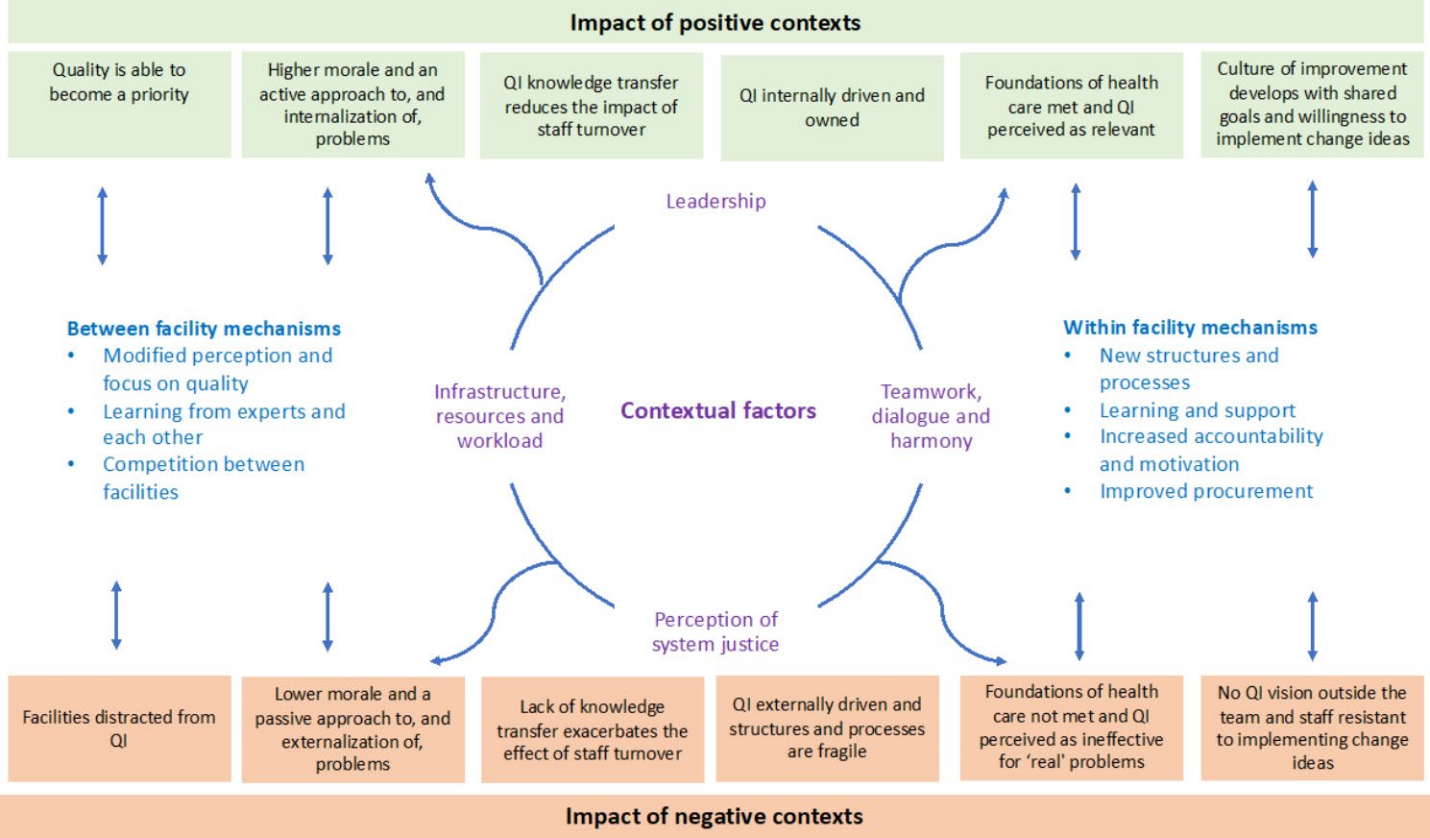
Contextual feedback loops and how they positively and negatively impact QI

The contexts described above did not occur in isolation but were linked and interacted with each other, these contexts had various impacts on QICs and on QIC mechanisms. Figure 1 shows the relationship between contextual factors and how these generated a positive enable context (shown in green) for QI, or conversely a negative context (shown in orange) and the mechanisms that these impacted on (shown in blue).

## Discussion

This qualitative study is one of the few exploring mechanisms of action and contextual factors affecting a scaled-up quality improvement intervention at health centre and hospital levels in a low-income setting. We identified QI mechanisms that worked between facilities: modified perceptions and focus on quality, learning from experts and each other, and competition between facilities; and mechanisms that worked within facilities: new structures and processes, learning and support, increased accountability and motivation, and improved procurement. The mechanisms we identified were broadly similar to those reported elsewhere [5, 7], however we found that some previously reported mechanisms such as improved teamwork, problem solving and shared leadership were enabling contextual issues in the study facilities rather than something that QI modified. The study confirms the need to carefully consider context in the planning and implementation of QICs [5–7]. However, identifying individual contextual factors that could be used as a signal that QI needs to be adapted is made difficult as contexts are fluid, with multiple interactions and linkages- a systems thinking approach to context may be more appropriate [16].

Mechanisms were more likely to be triggered in facilities with strong leaders, good teamwork and dialogue and sufficient resources and infrastructure – these were the facilities classified as higher performing in relation to QI. In region A the higher performing facility showed improvements in coverage indicators. Study region B was subject to political instability and civil unrest and neither the higher nor lower performing facility were able to achieve any improvements in coverage. The hospitals showed improvements in only one of the three coverage indicators and had more negative contextual environments than the higher performing health centres, but improvements in clinical management were a focus in hospitals and we do not have robust data on these outcomes. Others have found that QI in health centres can perform better than hospitals, which they attributed to more complex clinical processes, more limited engagement from leadership and high staff turnover in hospital settings [17].

We found that in facilities which lacked essential inputs, such as beds, equipment and drugs, staff struggled to see how QIC could meaningfully and sustainably improve quality and were less likely to have functioning and institutionalized QI; and an evaluation of the prototype phase of this project found greater improvement in facilities with better supplies [11]. It may be that in settings where the essential foundations for health care delivery are not met quality needs to be addressed at the system rather than the facility level [6], and whether QIC should include such facilities has been questioned [18, 19]. This stance is supported by other studies that highlight that staff shortages, staff turn-over, high work-loads and structural constraints limit a facility’s ability to deliver quality care and to successfully engage with QI [18–22]. But other studies have found that facilities in resource constrained settings are able to make sustained changes through QICs without material inputs or that QI empowered them to provide care despite structural challenges [17, 23]. Although some inputs may be essential for service delivery there is poor correlation between infrastructure and adherence to evidence-based guidelines [24], and the situation is likely to be more complex than a lack of infrastructure alone with neither inputs nor QIC sufficient to change quality alone in such settings.

The lower performing facilities in our study were characterised by weak leadership, conflict and a lack of teamwork which meant that a culture for improvement was not developed and QI structures and processes required external drivers in the form of the IHI mentor rather than being internalized and institutionalized. QIC being implemented at surface level rather than with authentic commitment has been recognized as a risk in other contexts [25, 26], and this resonates with our findings. The higher performing facilities had an enabling environment for QI and were active rather than passive in their approach to issues before the QIC. Our findings support the notion that facilities that implement QI successfully are most likely to be those that already have characteristics that foster quality and are organizationally ready [6, 27]. Facilities with more negative contexts will require support to improve their organizational culture. By design QIC runs for a limited time (6–15 months) [4] and the current commonly used time frames may be too short for the required changes in organizational culture- even where this is supported. QIC interventions in Nigeria and Rwanda extended their implementation period to reflect that QICs may need a greater time than anticipated to function [22, 28]. In some setting QICs have been adapted to be aligned with existing organizational cultures and have adopted top-down hierarchical decision making [22]. Whilst QICs need to adapted to the context they also need to maintain the core processes such as collaborative decision making, but may need support to do so.

As others have found there was a general enthusiasm for the QIC among those in the QI team, with both mentoring and the learning sessions identified as mechanisms for change and valued by participants. QI brought many positive changes to individuals and the facilities. Mentoring and the learning sessions were different to the regular *woreda* meetings which had a culture of blame for failures, and many aspects of the QI approach to learning and support would be beneficial to integrate into routine work structures. In some other settings QI mentoring was more valued than the learning sessions [23], but we found learning sessions provided a collaborative advantage over mentoring visits alone [26]. However, there were some challenges to positive collaboration during learning sessions in that presenting failure led to feelings of shame. This risk has been noted elsewhere [26], and in the study setting may be compounded by a historical culture of blame for poor performance [29, 30], which the QICs worked intentionally to change.

Collaboration and support outside of the QI team has been reported as important for success in QICs [31], but we found that this only occurred in facilities that already had a team culture of shared responsibilities. This team culture led to QI knowledge being shared, including when staff were replaced, and staff were willing to modify roles and tasks to help achieve improvement. In facilities without a team culture the QI structures and processes were sometimes alienating and poorly understood. This may be a function of the short QIC implementation period, but implementers should not assume that there will be organic QI knowledge transfer within facilities.

IHI mentors were key drivers of change within facilities as they provided opportunities for learning and support and through an accountability and motivation mechanism. Where QI was internalized the reliance on mentors reduced over time, but in the lower performing facilities QI remained externally driven. Other studies have identified that QI teams needed frequent external support to function [18, 32] and that government structures may not be able to provide this [32]. It is unsurprising that IHI and *woreda* mentors were viewed differently, but the transition to *woreda* mentors was a challenge for facilities as they felt less supported by *woreda* mentors, and a challenge for *woreda* mentors in terms of transport and workload. Further research is needed on the long-term sustainability of government mentors and how best to manage such transitions, but given their importance mentors need to be sufficient in number, have manageable workloads, skills and transport.

Health facilities in region B were affected for several months by the political instability and civil unrest of 2019. The study was not designed to explore the impact of conflict on quality, a topic that has received insufficient attention [33, 34], and understanding the complex political, social and economic nature and impact of conflict is beyond the scope of the study. The facilities in region B were weakened during the conflict through staffing and supply issues, closure, reduced support from community leaders and reduced uptake of services. In these context QI indicators degraded even in the higher performing facility, with positive contexts such as strong leadership not being able to act as a buffer at least in the short term. More information on the utility and resilience of QI in these settings is needed.

Our study had strengths and limitations that need to be considered when interpreting the findings. A strength of our study is the purposive selection of case studies allowed us to compare and contrast higher and lower performing facilities, and to compare health centres and hospitals. We limited the case-studies to six facilities to allow in-depth data collection and analysis, but this limits the transferability of our findings especially for the hospitals as only two were included. Facilities were selected to be similar to other facilities in their region which enhances the transferability of our findings, but the findings may not be transferable to settings with very different contexts. The interviews may have been subject to social desirability bias, although we trained our interviewers to reduce this by adopting a non-judgmental attitude towards respondents. We were limited in exploring whether improvement occurred through three coverage and one quality indicators, the study would have been strengthened if other quality indicators had been robust enough to use.

## Conclusion

We identified QI mechanisms that worked between and within facilities which were influenced by a set of interlinked contextual factors that together formed a positive or negative enabling environment. Facilities classed as higher performing in QI (i.e. where QI teams were active and QI was integrated into the workflow) had enabling environments and, in the absence political instability and civil unrest, showed improvement in coverage indicators. These facilities already had many of the characteristics that foster quality.

## Electronic supplementary material

Below is the link to the electronic supplementary material.

Supplementary Material 1

## Data Availability

The dataset generated and analysed during the current study are not publicly available as participants could be identified if their interviews are read in full but are available from the corresponding author on reasonable request.
