# Supplementary material for: “Quality teaches you how to use water. It doesn’t provide a water pump”: a qualitative study of context and mechanisms of action in an Ethiopian quality improvement program
Source: BMC Health Serv Res. 2023 Apr 19;23:381. doi: 10.1186/s12913-023-09341-7 (PMC10116784; doi:10.1186/s12913-023-09341-7)
Supplement: Supplementary file 1 — Supplementary Material 1 [file 12913_2023_9341_MOESM1_ESM.docx]

**Supplementary file 1: Clinical bundle definition**

| **Bundle name** | **Bundle definition** |
| --- | --- |
| Admission management | Danger sign assessment |
|  | Partograph initiated when cervical dilation at least 4 cm |
|  | Availability of soap, water, alcohol hand rub, and gloves |
|  | Birth companion encouraged to be present during labour and at birth |
|  | Mothers privacy maintained during labor and delivery |
| 2nd stage of labour | Availability of gloves, soap/savlon and clean water |
|  | Preparation of 10 IU IV/IM Oxytocin in syringe |
|  | Availability of two clean, dry, warm towels and suction device |
|  | Availability of bag and mask (size 0 and 1) |
|  | Helper/Assistant identified and informed for resuscitation |
| Post-delivery | Newborn assessment |
|  | Immediate skin to skin and initiate breastfeeding within the 1st hour |
|  | Baby weighed and recorded |
|  | Administer Vitamin K |
|  | Administer tetracycline eye ointment |

**Supplementary file 2 Control charts for four core improvement indicators for the study facilities**


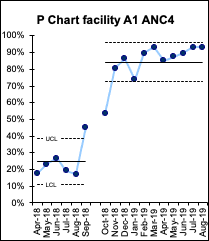

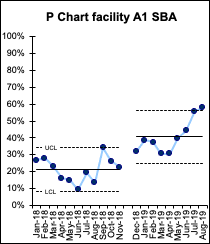

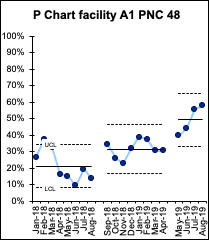

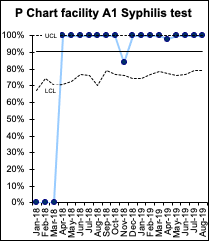


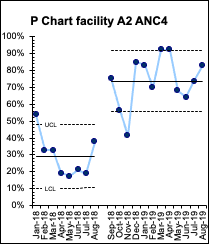

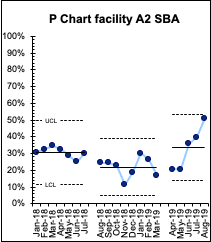

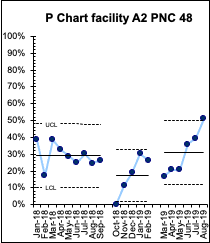

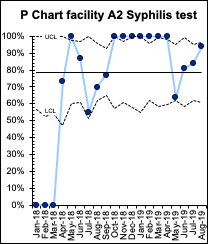


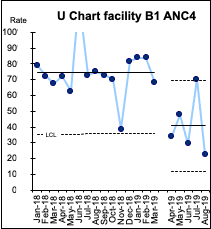

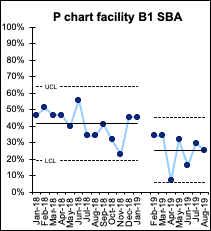

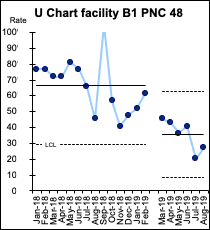

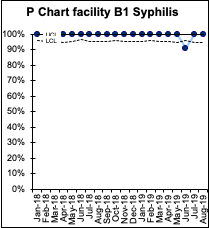


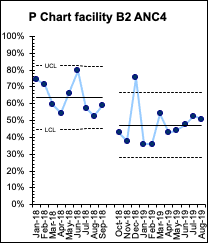

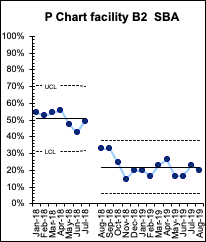

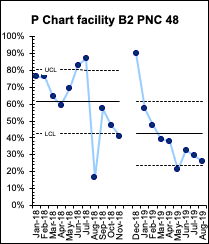

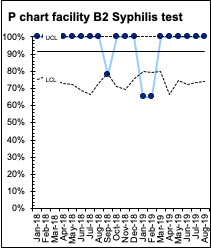


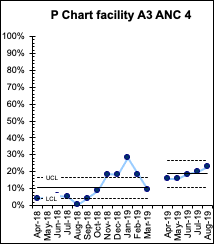

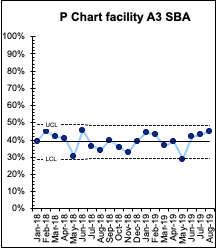

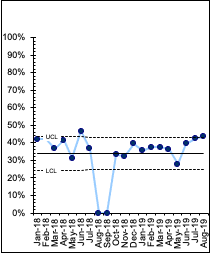

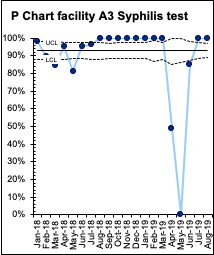


**P Chart facility A3 PNC 48**


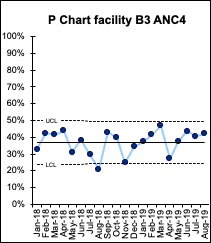

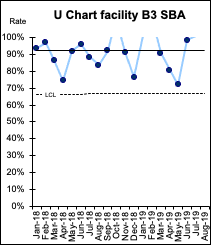

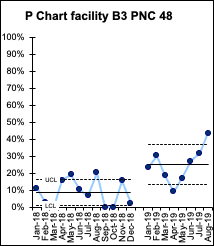

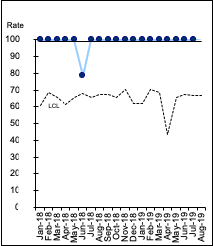


**U Chart facility B3 Syphilis test**

**Supplementary file 3: Summary of the QI core indicator P charts in relation to observed special cause changes^*^**

|  | **Health centres** | | | | **Hospitals** | |
| --- | --- | --- | --- | --- | --- | --- |
|  | **A1: High performing** | **A2: Low performing** | **B1: High performing** | **B2: Low performing** | **A3** | **B3** |
| **Coverage of ANC-4** | Improvement (Rule 4) | Improvement (Rule 4): outliers suggest instability (Rule 1) | Degradation after LS3 (small shift) outliers suggest instability (Rule 1) | Degradation after LS2 (Rule 3) outliers suggest instability (Rule 1) | Improvement after LS3 (Rule 2) | No change |
| **Coverage of SBA** | Improvement after LS3 (Rule 4) | Degradation after LS1 (Rule 3) and improvement after LS3 (Rule 2) | Degradation after LS3 (Rule 2) | Degradation after LS1 (Rule 3) | No change | No change |
| **Coverage of PNC-48** | Improvement (Rule 3) | Degradation after LS1 (Rule 2) and improvement after LS3 (Rule 5) | Degradation after LS3 (Rule 2) outliers suggest instability | Degradation after LS2 (Rule 5) outliers suggest instability (Rule 1) | No change, outliers suggest continued instability (Rule 1) | Improvement (Rule 3) outliers suggest instability (Rule 1) |
| **ANC attendees tested for syphilis** | High levels at baseline maintained | Improvement (Rule 4) outliers suggest continued instability (Rule 1) | High levels at baseline maintained | High levels at baseline maintained, outliers suggest instability (Rule 1) | High levels at baseline maintained, outliers suggest instability (Rule 1) | High levels at baseline maintained |

**^*^** Rule 1: Single data point outside the control limits, Rule 2: Four of five points above or below the central line, Rule 3: Eight or more data points above or below the central line, Rule 4: Two out of three consecutive points near the outer control limit, Rule 5: Six or more points increasing or decreasing
